# Adverse childhood experiences in adults completing ADHD and autism diagnostic assessment: associations with diagnostic outcome and psychiatric symptom burden

**DOI:** 10.3389/fpsyg.2026.1898116

**Published:** 2026-09-17

**Authors:** Marios Adamou, Sarah Louise Jones, Olivia Smith

**Affiliations:** 1 School of Human and Health Sciences, University of Huddersfield, Huddersfield, United Kingdom; 2 Adult ADHD and Autism Service, South West Yorkshire Partnership NHS Foundation Trust, Wakefield, United Kingdom

**Keywords:** ADHD, adult mental health, adverse childhood experiences, autism, childhood adversity, differential diagnosis, neurodevelopmental assessment, psychiatric symptom burden

## Abstract

**Background:**

Adult ADHD and autism assessment services increasingly receive clinically heterogeneous referrals in which the sequelae of childhood adversity may overlap with neurodevelopmental presentations. The distribution and clinical significance of adverse childhood experiences (ACEs) in this referral population remain insufficiently characterized.

**Methods:**

This retrospective cross-sectional cohort study analyzed routinely collected clinical data from 389 adults who attended either the ADHD or the autism diagnostic pathway of a regional National Health Service (NHS) Adult ADHD and Autism Service. The analytic sample consisted of 233 adults with a definitive diagnostic outcome at data extraction; 149 were awaiting an outcome, six had an inconclusive outcome, and one underage case was excluded. ACE exposure was measured with the 10-item ACE Questionnaire. Psychiatric symptom burden was assessed with routinely administered screening instruments (PHQ-9, GAD-7, AUDIT, DAST-10, MDQ, HELPS and IPDS). The primary analysis examined the association between ACE score, analyzed as a continuous variable, and diagnostic outcome, with adjustment for age, gender and assessment pathway in binary logistic regression; all other analyses were exploratory.

**Results:**

Forty-seven participants (20.2%) received a diagnosis of ADHD or autism and 186 (79.8%) did not meet DSM-5 criteria. The mean ACE score was 3.10 (SD = 2.59), and 39.7% of participants reported four or more ACEs, compared with 8.3% in an English national household survey. Participants who were not diagnosed reported higher ACE scores than participants who were diagnosed (3.37 vs. 2.04; Hedges *g* = 0.52, 95% CI 0.19–0.84, *p* = 0.002). In the adjusted model, each additional ACE was associated with lower odds of receiving a neurodevelopmental diagnosis (odds ratio 0.85, 95% CI 0.73–0.99, *p* = 0.040). The proportion diagnosed declined across ACE categories from 35.6% (no adversity) to 6.2% (six or more ACEs). ACE scores correlated positively with depressive symptoms (*r* = 0.37), anxiety symptoms (*r* = 0.28) and drug use scores (*r* = 0.27), but not alcohol use, and were higher among participants screening positive for possible personality disorder, head injury and mood disorder. In an exploratory model additionally adjusting for depressive symptoms and personality disorder screening status, the ACE association attenuated and was no longer statistically significant, indicating that it is not independent of concurrent psychiatric symptom burden.

**Conclusions:**

ACE exposure in this referral cohort was substantially higher than English population estimates and was associated with diagnostic non-confirmation and with greater psychiatric symptom burden. These findings support consideration of childhood adversity within trauma-informed formulation during adult neurodevelopmental assessment. Prospective studies incorporating direct measures of trauma-related psychopathology are needed to determine whether trauma-related mechanisms contribute to neurodevelopmental-like presentations. The cross-sectional retrospective design precludes causal inference.

## 1 Introduction

The volume of adult referrals for assessment of Attention Deficit Hyperactivity Disorder (ADHD) and autism spectrum disorder has increased substantially across healthcare systems, placing pressure on diagnostic services and extending waiting times. This increase appears to reflect a combination of improved recognition, broadened public awareness, changing help-seeking behavior, and service accessibility rather than a single local phenomenon (Berney, 2009; Silcock et al., 2025). Reports from NHS and international settings describe rising demand, fragmented provision and increasing reliance on independent or non-traditional assessment routes (Robinson et al., 2024; Smith et al., 2024; Raaj et al., 2024; Mendonsa and Jayasooriya, 2025; see also Maurice et al., 2022, on transition barriers).

Adult neurodevelopmental referral populations are clinically heterogeneous. Some people present with clear developmental histories and current functional impairment consistent with ADHD or autism. Others present with overlapping attentional, emotional, social, cognitive or behavioral difficulties that may arise from anxiety, depression, trauma, substance misuse, personality difficulty, acquired brain injury, or complex psychosocial adversity. This heterogeneity reduces the discriminatory value of symptom checklists when they are used without careful developmental and contextual formulation (Adamou et al., 2022, 2024; Jones et al., 2021).

High rates of psychiatric comorbidity further complicate assessment. Adults with ADHD, autism or combined presentations frequently report depressive, anxiety, substance use and other mental health difficulties (Crimlisk, 2011; Nylander et al., 2013). Comorbidity may coexist with a genuine neurodevelopmental condition, obscure developmental features, or produce symptoms that mimic ADHD or autism. For this reason, adult neurodevelopmental assessment requires more than symptom enumeration. It requires a temporal formulation that distinguishes lifelong developmental traits from later-emerging or context-dependent symptoms.

It would be useful to separate three constructs, as they are frequently conflated. The first is exposure to adverse childhood experiences (ACEs), a retrospective count of categories of childhood abuse, neglect and household dysfunction. The second is trauma-related psychopathology, such as post-traumatic stress disorder or complex post-traumatic stress disorder (PTSD), which requires current symptomatology and is not implied by exposure alone. The third is neurodevelopmental disorder, defined by developmental onset, pervasiveness and functional impairment. Exposure to adversity does not establish a trauma-related disorder, and neither does it exclude a neurodevelopmental condition. The present study measured the first construct only, and its findings must be interpreted within that constraint.

ACEs are established determinants of adult mental and physical health. The original ACE study demonstrated graded associations between childhood abuse, neglect and household dysfunction and multiple adult outcomes, including depression, substance misuse and functional impairment (Felitti et al., 1998). Subsequent studies and meta-analyses have replicated dose-response associations between ACE exposure and depression, anxiety, substance use, disability and health service utilization across different populations and methods (Chapman et al., 2004; Dube et al., 2003; Chang et al., 2019; van der Feltz-Cornelis et al., 2019; Gondek et al., 2021; Kim et al., 2021; Tan and Mao, 2023; Hendrix et al., 2023; Daníelsdóttir et al., 2024; Mugoya et al., 2024; Timmins et al., 2025), including anxiety outcomes specifically (Dahlan, 2025) and adult disability (Schüssler-Fiorenza Rose et al., 2014).

The clinical relevance of ACEs to adult ADHD and autism assessment is substantial because presentations that follow childhood adversity may resemble neurodevelopmental symptoms. Childhood adversity has been associated with attention difficulties, executive dysfunction, emotional dysregulation, social withdrawal, interpersonal mistrust, avoidance, hypervigilance, irritability and impaired occupational or relational functioning (Guidetti et al., 2023; Martínez et al., 2016; Weinstein et al., 2000). The corresponding differential diagnostic problem is well described in children, in whom trauma-related presentations and ADHD are readily confused in both directions (Stewart, 2015; Capolino, 2022; McCauley, 2024). These features overlap with common referral reasons for ADHD and autism assessment. The overlap does not imply that adversity-related difficulty and neurodevelopmental conditions are mutually exclusive. Rather, it creates a differential diagnostic problem: similar presenting symptoms may arise from different developmental mechanisms and may require different interventions.

The relationship between ACEs and neurodevelopmental conditions is also bidirectional and clinically complex. People with neurodevelopmental conditions may be at increased risk of bullying, social exclusion, exploitation, family stress and other adverse experiences. Gajwani and Minnis (2023) describe this as a form of double jeopardy, in which neurodevelopmental vulnerability and childhood adversity may compound each other (see also Hoover, 2020). Conversely, difficulties arising after adversity may lead to referral for neurodevelopmental assessment even when DSM-5 criteria are not ultimately met. This means that ACE screening is relevant both to differential diagnosis and to the recognition of co-occurring adversity in those who do receive a diagnosis.

Despite this clinical importance, ACE exposure in adult neurodevelopmental referral populations remains insufficiently described. Existing literature has addressed ACEs in general and psychiatric populations, and has considered symptom overlap between trauma, ADHD and autism, but there is limited service-level evidence examining whether ACE exposure differs by diagnostic outcome among adults referred for ADHD or autism assessment. This gap matters because diagnostic services are under pressure, and inaccurate or incomplete formulation can lead to inappropriate diagnostic labeling, missed trauma needs, or inefficient pathway use.

This study therefore examined ACE exposure in adults who completed diagnostic assessment in a regional NHS Adult ADHD and Autism Service. The aims were to describe the prevalence and distribution of ACEs in adults who attended and completed ADHD or autism diagnostic pathways; investigate whether ACE scores differed by diagnostic outcome; examine associations between ACE scores and psychiatric symptom measures; and explore demographic influences, including age and gender, on ACE reporting. The principal hypothesis, addressed by the primary analysis, was that higher ACE exposure would be associated with lower likelihood of receiving a neurodevelopmental diagnosis. Associations with psychiatric symptom burden and demographic variables were examined as exploratory secondary analyses.

## 2 Methods

### 2.1 Study design and setting

This was a retrospective cross-sectional cohort study using routinely collected clinical records from adults referred to a National Health Service (NHS) Adult ADHD and Autism Service in West Yorkshire between January 2025 and January 2026. The study is reported in accordance with the Strengthening the Reporting of Observational Studies in Epidemiology (STROBE) recommendations (von Elm et al., 2007); a completed STROBE checklist is provided as Supplementary material.

The service accepts referrals of adults aged 18 years or over from primary healthcare providers, secondary care services and social services. Self-referrals are not accepted. Referrals are triaged by experienced clinicians against service acceptance criteria, and accepted referrals are allocated to either the ADHD or autism diagnostic pathway based on the referral question. The two pathways operate as distinct assessment processes with condition-specific diagnostic instruments, but both draw on the same routinely administered screening battery, described below. During the study period, the approximate waiting time from referral to assessment was between 2 and 4 years for the ADHD pathway and up to 13 weeks for the autism pathway. Each participant in the analytic sample was assessed through a single pathway during the study period, and no participant received both an ADHD and an autism diagnosis within the study window.

### 2.2 Participants

The source cohort comprised 389 adults who attended either the ADHD (*n* = 239) or the autism (*n* = 150) diagnostic pathway during the study period. People under the age of 18 years and people with a diagnosed intellectual disability were excluded; one case aged under 18 years was identified in the extracted dataset and removed prior to analysis, leaving 388 eligible cases. At the point of data extraction, 149 cases (38.4%) had attended the pathway but had not yet received a diagnostic outcome, and six cases (1.5%) had an inconclusive outcome. The final analytic sample therefore comprised 233 cases (60.1% of eligible cases) with a definitive diagnostic outcome. Participant flow, including exclusions and available-case denominators, is shown in Figure 1.

**Figure 1:**
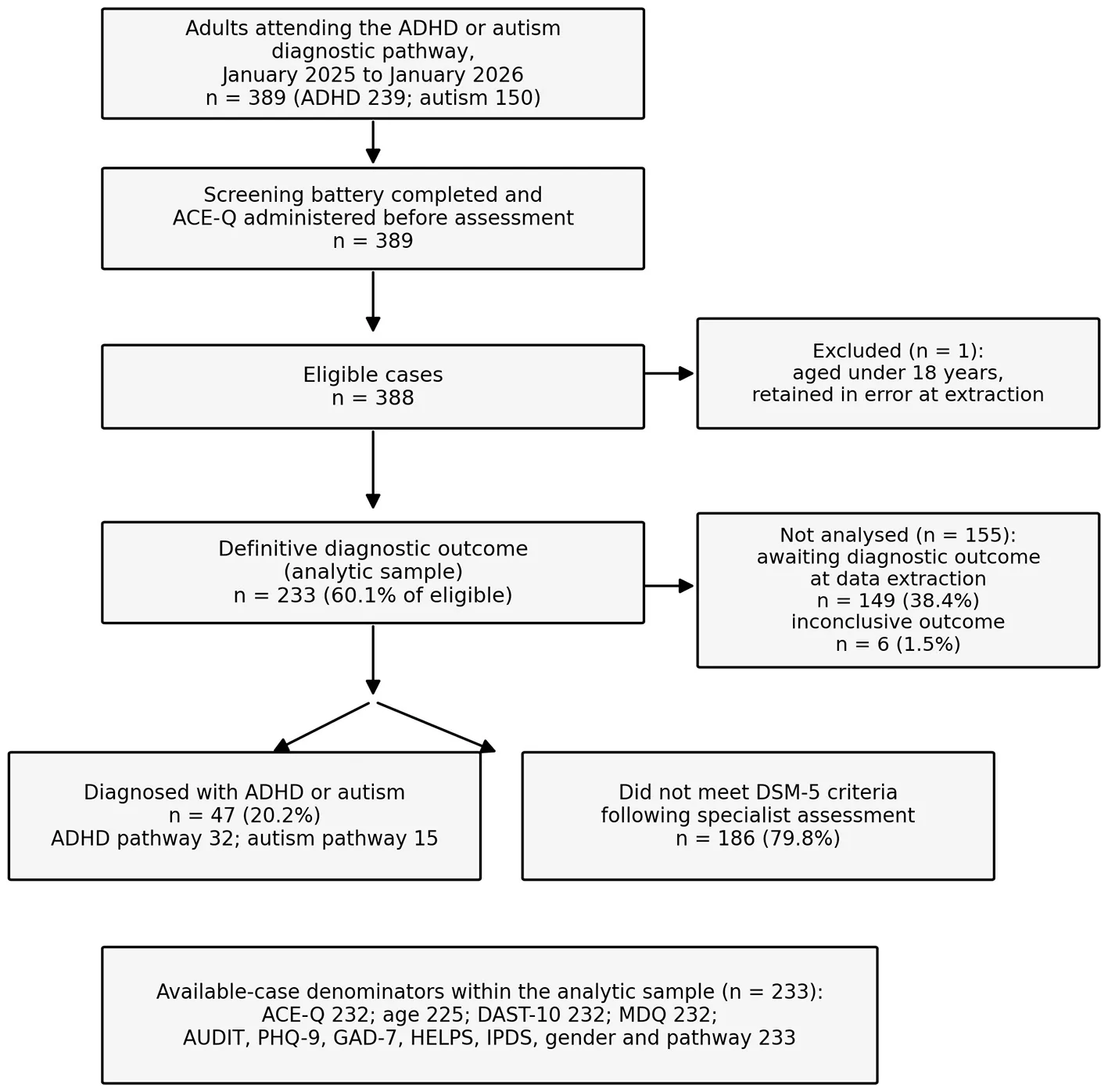
Participant flow through the study, with exclusions and available-case denominators.

Cases awaiting an outcome at extraction reflect assessments still in progress at the census date rather than disengagement from the service; inclusion in the analytic sample was therefore determined principally by the timing of assessment completion relative to data extraction. To assess the representativeness of the analytic sample, included cases were compared with cases awaiting an outcome on all available baseline characteristics (Table 1).

**Table 1:** Comparison of included cases and cases awaiting a diagnostic outcome.

Characteristic	Included (*n* = 233)	Awaiting outcome (*n* = 149)	Test statistic	*p*
Age, years, *M* (SD)	31.29 (11.54)	32.25 (10.88)	*t* = −0.79	0.43
Female, *n* (%)	126 (54.1%)	83 (55.7%)	χ^2^ = 0.04	0.84
ADHD pathway, *n* (%)	136 (58.4%)	99 (66.4%)	χ^2^ = 2.17	0.14
ACE-Q raw score, *M* (SD)	3.10 (2.59)	3.07 (2.72)	*t* = 0.13	0.90
AUDIT, *M* (SD)	4.92 (6.20)	4.80 (5.88)	*t* = 0.18	0.86
DAST-10, *M* (SD)	0.72 (1.68)	0.80 (2.01)	*t* = −0.44	0.66
PHQ-9, *M* (SD)	14.22 (6.58)	14.83 (6.96)	*t* = −0.85	0.39
GAD-7, *M* (SD)	12.15 (5.82)	12.94 (5.90)	*t* = −1.29	0.20
MDQ positive, *n* (%)	89/232 (38.4%)	66/143 (46.2%)	χ^2^ = 1.91	0.17
HELPS positive, *n* (%)	38/233 (16.3%)	20/149 (13.4%)	χ^2^ = 0.39	0.53
IPDS positive, *n* (%)	160/233 (68.7%)	111/149 (74.5%)	χ^2^ = 1.23	0.27

Comparison of the analytic sample with eligible cases awaiting a diagnostic outcome at data extraction. Denominators vary slightly with available data. Chi-square tests use Yates continuity correction.

Diagnostic outcome decisions were made in line with DSM-5 criteria by qualified and experienced multidisciplinary clinicians following full specialist assessment within a National Institute for Health and Care Excellence (NICE)-compliant NHS pathway. ADHD assessment included Diagnostic Interview for ADHD in Adults (DIVA-2) data, which incorporates informant and developmental history where available. Autism assessment included Adult Asperger Assessment (AAA) and Autism Diagnostic Observation Schedule, Second Edition (ADOS-2) assessment where clinically indicated; the AAA incorporates informant report. Cases were classified as diagnosed if they received an ADHD or autism diagnosis and not diagnosed if they did not meet DSM-5 criteria following specialist assessment. Diagnostic outcome was determined by the same multidisciplinary decision-making process in both pathways, applying the DSM-5 criteria relevant to each condition. Of the 47 diagnosed cases, 32 were diagnosed within the ADHD pathway and 15 within the autism pathway. The composite outcome of diagnosis of ADHD or autism was adopted because the study question concerned confirmation or non-confirmation of a neurodevelopmental diagnosis at service level; pathway-specific analyses are reported separately.

### 2.3 Measures

As part of routine service operation, screening questionnaires were distributed and completed before appointments to ensure that relevant clinical information was available to inform assessment. The ACE Questionnaire was therefore completed before diagnostic assessment, and its responses, together with the other screening instruments, were available to assessing clinicians as part of the routine clinical record. The implications of this for interpretation are considered in the Discussion. Support was available for patients who required assistance completing questionnaires.

#### 2.3.1 Generalized Anxiety Disorder Assessment (GAD-7)

The GAD-7 is a seven-item screening instrument for generalized anxiety symptoms, with total scores ranging from 0 to 21. Scores of 5–9, 10–14 and 15 or above indicate mild, moderate and severe anxiety symptom ranges, respectively (Spitzer et al., 2006; Löwe et al., 2008). The instrument is recommended for the identification of anxiety symptoms in United Kingdom practice (NICE, 2011).

#### 2.3.2 Patient Health Questionnaire (PHQ-9)

The PHQ-9 is a nine-item depression screening instrument based on DSM-IV symptom criteria. Scores range from 0 to 27, with higher scores indicating greater depressive symptom burden (Kroenke et al., 2001; Arroll et al., 2010; Manea et al., 2015).

#### 2.3.3 Mood Disorder Questionnaire (MDQ)

The MDQ screens for bipolar spectrum symptoms in outpatient settings. A positive screen requires endorsement of at least seven symptoms, symptom co-occurrence and moderate or serious impairment (Hirschfeld et al., 2000). A positive screen indicates possible bipolar spectrum symptomatology and is not equivalent to a diagnosis of bipolar disorder.

#### 2.3.4 Alcohol Use Disorders Identification Test (AUDIT)

The AUDIT is a 10-item World Health Organization screening instrument for hazardous and harmful alcohol use. Total scores range from 0 to 40, with higher scores indicating greater alcohol-related risk (Saunders et al., 1993).

#### 2.3.5 Drug Abuse Screening Test 10-item version (DAST-10)

The DAST-10 is a 10-item screening instrument for drug-related problems. Scores indicate none, low, moderate, substantial or severe drug-related difficulty (Skinner, 1982; Yudko et al., 2007).

#### 2.3.6 HELPS traumatic brain injury screen

The HELPS screen identifies possible traumatic brain injury through questions concerning head injury, emergency care, loss of consciousness, persisting problems and sickness symptoms. Three or more positive responses suggest possible brain injury requiring clinical consideration (Picard et al., 1991). A positive screen is not equivalent to confirmed traumatic brain injury.

#### 2.3.7 Iowa Personality Disorder Screen (IPDS)

The IPDS is an 11-item personality disorder screening instrument. A total score of three or more indicates a positive screen for possible personality disorder (Langbehn et al., 1999; Germans et al., 2010). A positive screen is not equivalent to a diagnosis of personality disorder.

#### 2.3.8 Adverse Childhood Experience Questionnaire (ACE-Q)

The ACE-Q is a 10-item instrument assessing exposure to childhood abuse and household dysfunction. Each endorsed item scores one point, producing a total score from 0 to 10 (Felitti et al., 1998). The ACE-Q measures exposure to categories of adversity; it does not measure severity, timing, chronicity, subjective impact or current trauma-related symptomatology. The primary analysis therefore used the raw ACE score as a continuous variable. For descriptive presentation, scores were additionally grouped into four categories: no reported adversity (0), low to moderate adversity (1–3), high adversity (4–5), and very high adversity (6 or more). The threshold of four or more ACEs derives from the original ACE study, in which risks for multiple adult outcomes rose markedly at this level of exposure (Felitti et al., 1998), and has been used in United Kingdom population surveys (Bellis et al., 2014), and the same boundaries of 0, 1–3 and 4 or more are used for clinical risk stratification in guidance on the clinical use of the ACE tool (ACEs Aware, 2019); the further subdivision at six or more was adopted descriptively in this study to characterize the upper tail of exposure and was not treated as an inferential threshold.

### 2.4 Ethical and governance considerations

The data were collected retrospectively as part of routine clinical operations. The project was reviewed by the service governance team, which determined that it constituted a service assessment using anonymised routine data and that formal research ethics committee approval was not required under the Health Research Authority decision framework. Patients were assessed in accordance with national clinical guidance, and information was handled in accordance with the Data Protection Act 2018 and the common law duty of confidentiality. Patients were informed through an opt-out process during initial clinical intake that anonymised clinical data could be used for service assessment and research purposes; this process operates alongside and is consistent with the NHS national data opt-out. All study data were anonymised before analysis.

### 2.5 Statistical analysis

Analyses were conducted using SPSS version 31 (IBM Corp., Armonk, NY, United States), with verification of the revised analyses in Python (statsmodels and SciPy). Descriptive statistics were used to summarize demographic, clinical and diagnostic characteristics. Continuous variables were summarized using means and standard deviations; categorical variables were summarized using frequencies and percentages. Available-case analysis was used for each statistical test. Missing data were few: within the analytic sample, age was missing for eight cases (3.4%), and ACE-Q, DAST-10 and MDQ scores were each missing for one case (0.4%); all other variables were complete. Denominators are reported for every analysis, and the association between missingness and diagnostic outcome, gender and pathway was examined. Because ACE data were near-complete (99.6%), missingness in the exposure cannot materially bias the reported associations; the more consequential completeness issue, the proportion of the source cohort awaiting a diagnostic outcome, is addressed through the included-versus-awaiting comparison in Table 1 and in the Discussion.

The association between ACE score and diagnostic outcome was designated the primary analysis. It was tested first by comparing ACE scores between diagnosed and non-diagnosed groups, and then in a binary logistic regression with diagnostic outcome as the dependent variable and ACE raw score as a continuous predictor, adjusting for age, gender and assessment pathway. This covariate set was restricted to three prespecifiable demographic and design variables to maintain approximately ten or more events per predictor with 47 diagnosed cases. Odds ratios with 95% confidence intervals are reported, together with a likelihood ratio test for the contribution of ACE score and multicollinearity diagnostics. An additional exploratory model added PHQ-9 score and IPDS screening status to examine whether the ACE association was independent of concurrent psychiatric symptom burden; because this model contains six predictors for 46 events, it is interpreted as exploratory only. A sensitivity analysis repeated the primary model with the six inconclusive cases coded as non-diagnosed.

All other analyses were exploratory secondary analyses and are interpreted accordingly, with emphasis on effect sizes and confidence intervals rather than isolated *p*-values. Independent-samples *t*-tests compared continuous variables between binary groups, using Welch's correction where Levene's test indicated unequal variances. Pearson correlations examined associations between ACE raw scores and continuous clinical measures, with 95% confidence intervals derived from the Fisher transformation. Because PHQ-9 and GAD-7 scores were strongly correlated (*r* = 0.78), their separate associations with ACE scores are not interpreted as independent effects. Chi-square tests examined associations between ACE categories and categorical outcomes, with Cramer's V and bootstrap 95% confidence intervals to estimate the strength of association; a Cochran–Armitage test examined the trend in diagnosis rate across ordered ACE categories. Where an expected cell count fell below five, a Monte Carlo exact *p*-value (100,000 tables sampled with fixed margins) was computed alongside the asymptotic test, and pathway specificity was tested formally by adding an ACE-by-pathway interaction term to the primary logistic model. One-way analysis of variance was used to examine differences in psychiatric symptom scores across ACE categories, with Bonferroni-adjusted *post hoc* comparisons. Multiple linear regression was used to examine whether age and gender predicted the ACE raw score. Gender was recorded in the clinical record as a binary administrative variable (male or female) and is described as gender throughout. Statistical significance was set at *p* < 0.05. No formal correction for multiple testing was applied across the family of secondary analyses; these analyses are explicitly labeled exploratory, and findings are interpreted according to magnitude, consistency and clinical plausibility rather than statistical significance alone.

## 3 Results

### 3.1 Sample characteristics and missing data

Participant flow is shown in Figure 1. The final analytic sample consisted of 233 participants with a definitive diagnostic outcome. Of these, 126 (54.1%) were female and 107 (45.9%) were male. The mean age was 31.29 years (SD = 11.54; range 18–76; *n* = 225). One hundred and thirty-six participants (58.4%) completed the ADHD pathway and 97 (41.6%) completed the autism pathway. Forty-seven participants (20.2%) received a diagnosis (32 ADHD; 15 autism) and 186 (79.8%) did not meet DSM-5 diagnostic criteria. Within the ADHD pathway, 32 of 136 (23.5%) received a diagnosis, compared with 15 of 97 (15.5%) within the autism pathway; this pathway difference was not statistically significant, $\chi_{\left(1\right)}^{2}$ = 1.814, *p* = 0.178. Men were more likely to be diagnosed than women (33/107, 30.8%, vs. 14/126, 11.1%). Participant characteristics are summarized in Table 2.

**Table 2:** Participant demographics and clinical characteristics (*N* = 233).

Characteristic	*n*	Mean (SD) or *n* (%)	Range
Age, years	225	31.29 (11.54)	18–76
Female	233	126 (54.1%)	—
Male	233	107 (45.9%)	—
ADHD pathway	233	136 (58.4%)	—
Autism pathway	233	97 (41.6%)	—
Diagnosed with ADHD or autism	233	47 (20.2%)	—
Diagnosed, ADHD pathway	136	32 (23.5%)	—
Diagnosed, autism pathway	97	15 (15.5%)	—
Not diagnosed	233	186 (79.8%)	—
ACE-Q raw score	232	3.10 (2.59)	0–10
No reported adversity (ACE = 0)	232	45 (19.4%)	—
Low to moderate adversity (ACE = 1–3)	232	95 (40.9%)	—
High adversity (ACE = 4–5)	232	44 (19.0%)	—
Very high adversity (ACE ≥ 6)	232	48 (20.7%)	—
AUDIT alcohol use	233	4.92 (6.20)	0–30
DAST-10 drug use	232	0.72 (1.68)	0–10
PHQ-9 depression	233	14.22 (6.58)	0–27
GAD-7 anxiety	233	12.15 (5.82)	0–21
MDQ positive screen	232	89 (38.4%)	—
HELPS positive screen	233	38 (16.3%)	—
IPDS positive screen	233	160 (68.7%)	—

The *n* column gives the available-case denominator for each variable within the analytic sample of 233. Positive screening status indicates possible clinical concern requiring further assessment, not a confirmed diagnosis. ADHD, Attention Deficit Hyperactivity Disorder; ACE, Adverse Childhood Experiences; AUDIT, Alcohol Use Disorders Identification Test; DAST-10, Drug Abuse Screening Test; PHQ-9, Patient Health Questionnaire-9; GAD-7, Generalized Anxiety Disorder-7; MDQ, Mood Disorder Questionnaire; HELPS, head injury screening tool; IPDS, Iowa Personality Disorder Screen.

Missing data were minimal. Age was missing for eight participants (3.4%); ACE-Q, DAST-10 and MDQ scores were each missing for one participant (0.4%); all other variables were complete. Age missingness was not associated with diagnostic outcome (Fisher exact *p* = 0.364) or gender (*p* = 0.729) but was associated with pathway, with all eight missing values occurring in the autism pathway (*p* = 0.001). Because ACE data were available for 232 of 233 participants, exposure missingness cannot materially affect the reported associations.

Included participants did not differ significantly from the 149 cases awaiting a diagnostic outcome on any measured baseline characteristic, including age, gender, pathway, ACE score, or any psychiatric screening measure (Table 1; all p >0.13). Cases awaiting an outcome reflect assessments in progress at the census date rather than disengagement.

### 3.2 Demographic influences on ACE scores (exploratory)

Female participants reported higher ACE raw scores (*M* = 3.48, SD = 2.50, *n* = 126) than male participants (*M* = 2.66, SD = 2.65, *n* = 106), *t*_(230)_ = 2.410, *p* = 0.017, *d* = 0.32. ACE raw score showed a small positive association with age, *r* = 0.17 (95% CI 0.04–0.29), *p* = 0.013, *n* = 224.

A multiple linear regression model predicting ACE raw score from age and gender (*n* = 224) showed independence of residuals (Durbin–Watso *n* = 1.859). The model accounted for a small but statistically significant proportion of variance, *R*^2^ = 0.045, adjusted *R*^2^ = 0.036, *F*_(2, 221)_ = 5.220, *p* = 0.006. Age made a small independent contribution (B = 0.035, 95% CI 0.006–0.064, *p* = 0.020); the contribution of gender was smaller and its confidence interval approached zero at the lower bound (B = 0.695, 95% CI 0.017–1.374, *p* = 0.045), and it should be interpreted cautiously. Full coefficients are reported in Table 3.

**Table 3:** Multiple linear regression model predicting ACE raw score.

Predictor	*B*	SE *B*	95% CI for *B*	β	*p*
Constant	1.599	0.517	0.580–2.619	–	0.002
Age (years)	0.035	0.015	0.006–0.064	0.155	0.020
Gender (female = 1)	0.695	0.344	0.017–1.374	0.133	0.045

Dependent variable: ACE-Q raw score (*n* = 224 with complete age and ACE data).

B, unstandardised regression coefficient; SE B, standard error of B; CI, confidence interval; β, standardized coefficient.

Gender was recorded as a binary administrative variable and coded male = 0, female = 1. Model: *R*^2^ = 0.045, adjusted *R*^2^ = 0.036, F_(2, 221)_ = 5.220, *p* = 0.006.

### 3.3 ACE exposure and diagnostic outcome (primary analysis)

Participants who did not receive a neurodevelopmental diagnosis reported higher ACE scores (*M* = 3.37, SD = 2.64, *n* = 186) than participants who were diagnosed (*M* = 2.04, SD = 2.12, *n* = 46), *t*_(230)_ = 3.153, *p* = 0.002, Hedges *g* = 0.52 (95% CI 0.19–0.84); the result was unchanged with Welch's correction (*t* = 3.598, *p* < 0.001) and on non-parametric testing (Mann–Whitney *U, p* = 0.002).

In unadjusted logistic regression, each additional ACE was associated with lower odds of receiving a diagnosis, odds ratio (OR) 0.79 (95% CI 0.68–0.92), *p* = 0.003 (*n* = 232). In the primary adjusted model including age, gender and assessment pathway (*n* = 224), the association persisted, OR 0.85 per ACE point (95% CI 0.73–0.99), *p* = 0.040; likelihood ratio test for ACE, χ^2^(1) = 4.598, *p* = 0.032; Nagelkerke *R*^2^ = 0.174. Female gender was independently associated with lower odds of diagnosis (OR 0.32, 95% CI 0.15–0.66, *p* = 0.002); age and pathway were not statistically significant. All variance inflation factors were below 1.1. A sensitivity analysis coding the six inconclusive cases as non-diagnosed produced a materially identical ACE estimate (OR 0.86, 95% CI 0.74–1.00, *p* = 0.044). Full models are reported in Table 4.

**Table 4:** Logistic regression models predicting diagnostic outcome.

Predictor	OR	95% CI	*p*
Unadjusted model (*n* = 232)			
ACE-Q raw score (per point)	0.79	0.68–0.92	0.003
Primary adjusted model (*n* = 224)			
ACE-Q raw score (per point)	0.85	0.73–0.99	0.040
Age (per year)	0.97	0.93–1.00	0.059
Gender (female = 1)	0.32	0.15–0.66	0.002
Pathway (ADHD = 1)	1.67	0.80–3.47	0.170
Exploratory model (*n* = 224; interpret with caution)			
ACE-Q raw score (per point)	0.91	0.77–1.07	0.246
Age (per year)	0.97	0.93–1.00	0.047
Gender (female = 1)	0.34	0.16–0.71	0.004
Pathway (ADHD = 1)	1.73	0.82–3.63	0.149
PHQ-9 (per point)	0.96	0.90–1.02	0.217
IPDS positive screen	0.76	0.35–1.67	0.494

Note. Dependent variable: diagnosis of ADHD or autism (diagnosed = 1). Primary adjusted model: likelihood ratio test for ACE, χ^2^(1) = 4.598, *p* = 0.032; Nagelkerke *R*^2^ = 0.174; all variance inflation factors < 1.1. The exploratory model contains six predictors for 46 events and is reported for transparency only.

OR, odds ratio; CI, confidence interval.

In an exploratory model additionally adjusting for PHQ-9 score and IPDS screening status (six predictors, 46 events), the ACE estimate attenuated toward the null and was no longer statistically significant (OR 0.91, 95% CI 0.77–1.07, *p* = 0.246). This model is underpowered relative to conventional events-per-predictor guidance and is reported for transparency; it indicates that the association between ACE exposure and diagnostic non-confirmation is not independent of concurrent psychiatric symptom burden, a point taken up in the Discussion.

Descriptively, the proportion of participants receiving a diagnosis declined monotonically across ACE categories: 16 of 45 (35.6%) with no reported adversity, 19 of 95 (20.0%) with low to moderate adversity, eight of 44 (18.2%) with high adversity, and three of 48 (6.2%) with very high adversity (Cochran–Armitage trend test, *z* = −3.36, *p* < 0.001; Figure 2). ACE category was associated with diagnostic outcome, $\chi_{\left(3\right)}^{2}$ = 12.646, *p* = 0.006, *V* = 0.23 (bootstrap 95% CI 0.14–0.37). Very high adversity was reported by 45 of 186 (24.2%) non-diagnosed participants compared with three of 46 (6.5%) diagnosed participants, whereas no adversity was reported by 16 of 46 (34.8%) diagnosed participants compared with 29 of 186 (15.6%) non-diagnosed participants. The full distribution with cell counts is shown in Figure 3.

**Figure 2:**
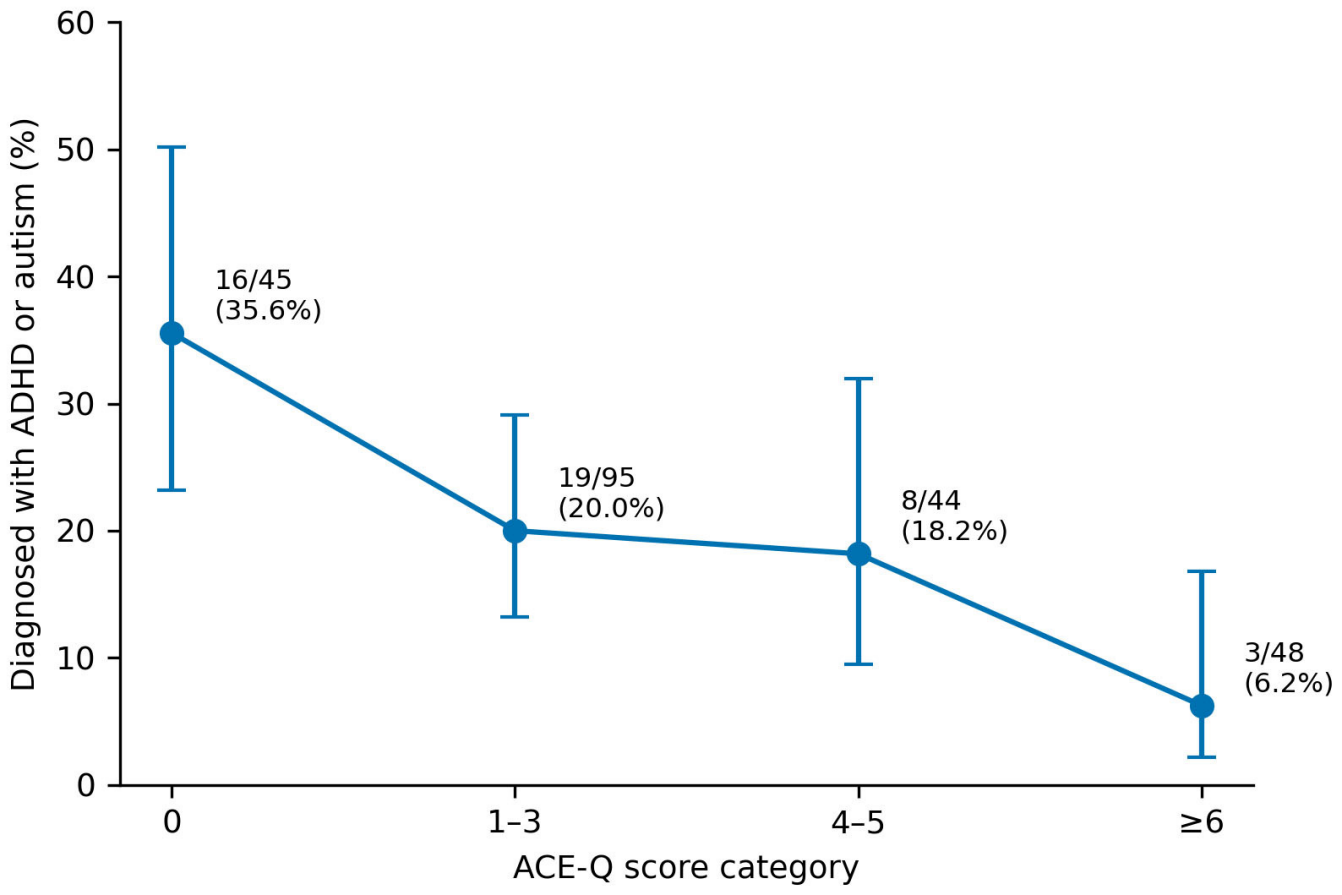
Proportion of participants receiving an ADHD or autism diagnosis by ACE-Q score category, with 95% Wilson confidence intervals. Labels give counts of diagnosed participants over category totals.

**Figure 3:**
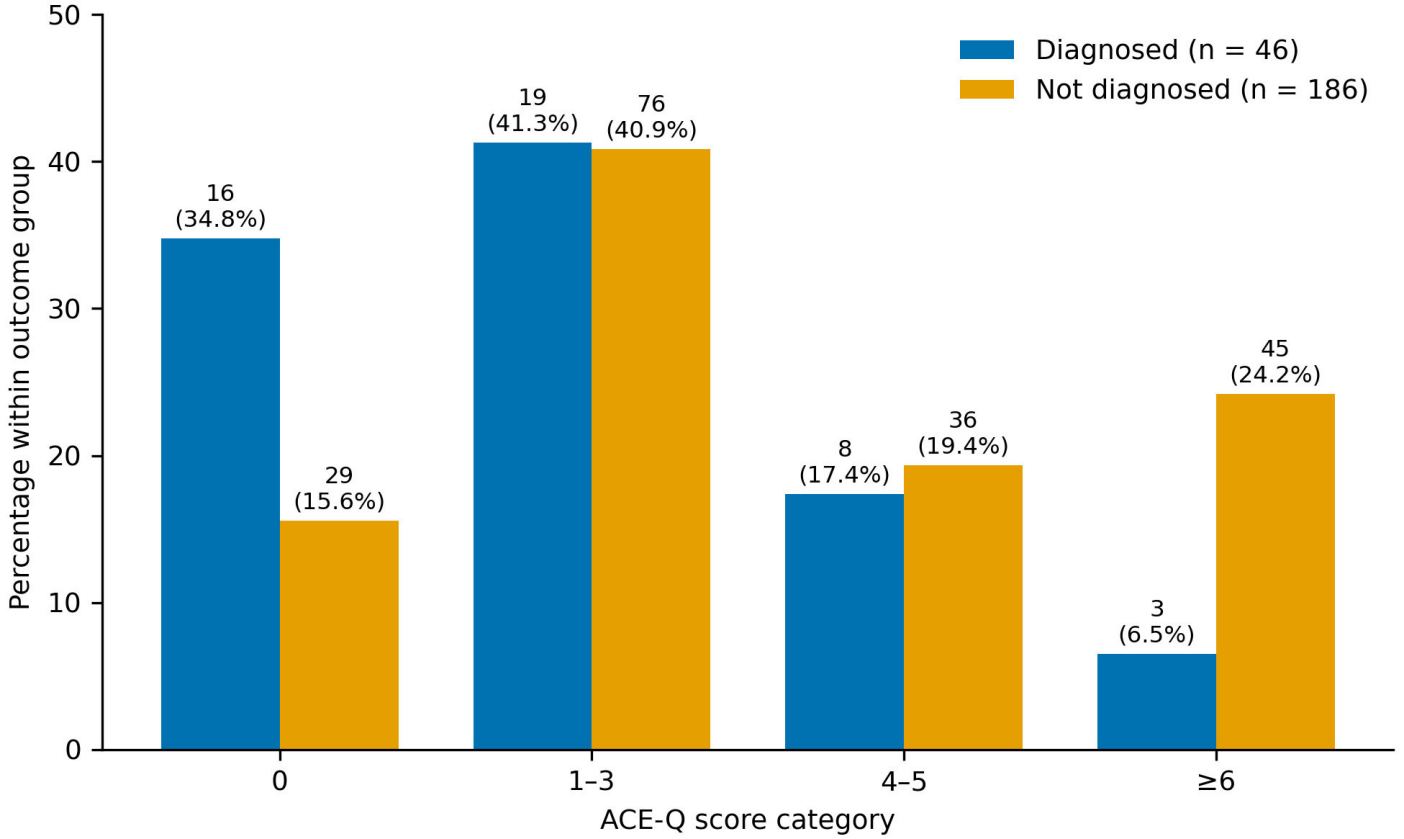
ACE-Q score category distribution by diagnostic outcome. Bars show percentages within each outcome group; labels give cell counts and percentages. Diagnosed *n* = 46 and not diagnosed *n* = 186 (one diagnosed participant had no ACE-Q data).

Pathway-specific analyses were consistent in direction and magnitude across the two pathways when ACE score was analyzed continuously: within the ADHD pathway, diagnosed participants reported lower ACE scores than non-diagnosed participants (*M* = 2.13, *n* = 31, vs. *M* = 3.20, *n* = 104; Welch *t* = 2.459, *p* = 0.016), as they did within the autism pathway (*M* = 1.87, *n* = 15, vs. *M* = 3.57, *n* = 82; Welch *t* = 2.397, *p* = 0.027). On the categorical analysis, the association reached statistical significance within the autism pathway, $\chi_{\left(3\right)}^{2}$ = 9.061, *p* = 0.029, *V* = 0.31 (95% CI 0.14–0.54), but not within the ADHD pathway, $\chi_{\left(3\right)}^{2}$ = 5.515, *p* = 0.138, *V* = 0.20 (95% CI 0.11–0.37). Because one expected cell count in the autism pathway was below five, Monte Carlo exact *p*-values were computed and were essentially identical to the asymptotic values (autism *p* = 0.028; ADHD *p* = 0.132; whole cohort *p* = 0.005). A formal test of pathway specificity, adding an ACE-by-pathway interaction term to the primary adjusted model, showed no evidence of a differential association (interaction odds ratio 1.19, 95% CI 0.86–1.66; likelihood ratio $\chi_{\left(1\right)}^{2}$ = 1.14, *p* = 0.285). Given the small subgroup sizes, overlapping confidence intervals, the consistency of the continuous analyses and the non-significant interaction, these results provide no reliable evidence that the association is specific to either pathway.

### 3.4 ACE associations with psychiatric symptom burden (exploratory)

ACE raw scores demonstrated statistically significant positive correlations with depressive symptoms, anxiety symptoms and drug use scores. ACE raw score showed a moderate positive association with PHQ-9 scores, *r* = 0.37 (95% CI 0.26–0.48), *p* < 0.001, and small positive associations with GAD-7 scores, *r* = 0.28 (95% CI 0.16–0.40), *p* < 0.001, and DAST-10 scores, *r* = 0.27 (95% CI 0.15–0.39), *p* < 0.001. No statistically significant association was observed between ACE raw score and AUDIT scores, *r* = −0.03 (95% CI −0.16 to 0.10), *p* = 0.63. PHQ-9 and GAD-7 scores were strongly intercorrelated (*r* = 0.78), and their separate associations with ACE scores should not be interpreted as independent effects. The full correlation matrix is shown in Table 5.

**Table 5:** Correlation matrix for ACE raw scores and continuous clinical measures.

Variable	1	2	3	4	5	*M*	SD	*n*
1. ACE-Q raw score	1	−0.03	0.27^***^	0.37^***^	0.28^***^	3.10	2.59	232
2. AUDIT	−0.03	1	0.35^***^	0.09	0.12	4.92	6.20	233
3. DAST-10	0.27^***^	0.35^***^	1	0.31^***^	0.18^**^	0.72	1.68	232
4. PHQ-9	0.37^***^	0.09	0.31^***^	1	0.78^***^	14.22	6.58	233
5. GAD-7	0.28^***^	0.12	0.18^**^	0.78^***^	1	12.15	5.82	233

Pearson correlations, available-case analysis. 95% confidence intervals for the ACE correlations are reported in the text.

^**^p < 0.01.

^***^p < 0.001.

Participants who screened positive on the MDQ reported higher ACE raw scores (*M* = 3.74, SD = 2.92, *n* = 88) than those who screened negative (*M* = 2.73, SD = 2.30, *n* = 143), Welch *t*_(152.5)_ = 2.762, *p* = 0.007, *g* = 0.39 (95% CI 0.13–0.66). Participants who screened positive on the HELPS reported higher ACE scores (*M* = 4.11, SD = 2.56, *n* = 38) than those who screened negative (*M* = 2.91, SD = 2.56, *n* = 194), *t*_(230)_ = 2.636, *p* = 0.009, *g* = 0.47 (95% CI 0.12–0.82). Participants who screened positive on the IPDS reported substantially higher ACE scores (*M* = 3.66, SD = 2.63, *n* = 159) than those who screened negative (*M* = 1.89, SD = 2.07, *n* = 73), Welch *t*_(174.2)_ = 5.537, *p* < 0.001, *g* = 0.72 (95% CI 0.43–1.00).

One-way analysis of variance (ANOVA) showed that ACE category was associated with DAST-10, PHQ-9 and GAD-7 scores, but not AUDIT scores. Drug use scores differed across ACE categories, *F*_(3, 227)_ = 4.911, *p* = 0.003, η^2^ = 0.06, with the clearest Bonferroni-adjusted difference between the no adversity and very high adversity categories (*p* = 0.027). Depressive symptom scores increased across ACE categories, *F*_(3, 228)_ = 14.300, *p* < 0.001, η^2^ = 0.16. Anxiety symptom scores also differed by ACE category, *F*_(3, 228)_ = 7.392, *p* < 0.001, η^2^ = 0.09, with the clearest difference between no adversity and very high adversity (*p* < 0.001). AUDIT scores did not differ by ACE category, *F*_(3, 228)_ = 0.246, *p* = 0.86, η^2^ = 0.003. These results are summarized in Table 6.

**Table 6:** Mean psychiatric symptom scores by ACE category.

Measure	No adversity *M* (SD), *n*	Low/moderate *M* (SD), *n*	High *M* (SD), *n*	Very high *M* (SD), *n*	*F*	*p*	η^2^
AUDIT	5.53 (6.55), 45	5.01 (5.85), 95	4.52 (6.27), 44	4.62 (6.64), 48	0.246	0.86	0.003
DAST-10	0.24 (0.65), 45	0.63 (1.29), 94	0.57 (1.35), 44	1.48 (2.77), 48	4.911	0.003	0.061
PHQ-9	9.80 (5.43), 45	14.35 (6.16), 95	14.36 (6.76), 44	18.06 (5.88), 48	14.300	< 0.001	0.158
GAD-7	9.09 (5.85), 45	12.52 (5.38), 95	11.91 (6.16), 44	14.48 (5.27), 48	7.392	< 0.001	0.089

ACE, Adverse Childhood Experiences. ACE categories: no reported adversity (0), low to moderate adversity (1–3), high adversity (4–5), very high adversity (6 or more). Exploratory analyses; Bonferroni-adjusted *post hoc* comparisons reported in the text.

Chi-square analyses showed that higher ACE category was associated with increased likelihood of screening positive on the IPDS, $\chi_{\left(3\right)}^{2}$ = 21.481, *p* < 0.001, *V* = 0.30 (bootstrap 95% CI 0.21–0.42), and on the HELPS, $\chi_{\left(3\right)}^{2}$ = 11.303, *p* = 0.010, *V* = 0.22 (95% CI 0.19–0.31); no participant with an ACE score of zero screened positive on the HELPS. The MDQ showed the same directional pattern but was not statistically significant, $\chi_{\left(3\right)}^{2}$ = 5.634, *p* = 0.131, *V* = 0.16 (95% CI 0.07–0.30). Cell counts are shown in Table 7.

**Table 7:** ACE category distribution by screening status.

Screen and status	No adversity *n* (%)	Low/moderate *n* (%)	High *n* (%)	Very high *n* (%)	χ^2^(3)	*p*	*V*
MDQ positive (*n* = 88)	16 (18.2%)	30 (34.1%)	17 (19.3%)	25 (28.4%)	5.634	0.131	0.16
MDQ negative (*n* = 143)	29 (20.3%)	64 (44.8%)	27 (18.9%)	23 (16.1%)	–	–	–
IPDS positive (*n* = 159)	22 (13.8%)	59 (37.1%)	36 (22.6%)	42 (26.4%)	21.481	< 0.001	0.30
IPDS negative (*n* = 73)	23 (31.5%)	36 (49.3%)	8 (11.0%)	6 (8.2%)	–	–	–
HELPS positive (*n* = 38)	0 (0.0%)	18 (47.4%)	9 (23.7%)	11 (28.9%)	11.303	0.010	0.22
HELPS negative (*n* = 194)	45 (23.2%)	77 (39.7%)	35 (18.0%)	37 (19.1%)	–	–	–

Percentages are row percentages within screening status. All expected cell counts were above five. Positive screening status indicates possible clinical concern, not a confirmed diagnosis. Exploratory analyses. MDQ, Mood Disorder Questionnaire; IPDS, Iowa Personality Disorder Screen; HELPS, head injury screening tool.

## 4 Discussion

### 4.1 Summary of principal findings

This retrospective cross-sectional cohort study examined adverse childhood experiences in adults who completed ADHD or autism diagnostic assessment in a regional NHS adult neurodevelopmental service. Four findings are important. First, ACE exposure was substantially higher than English population estimates, with 39.7% of participants reporting four or more ACEs compared with 8.3% in a national household survey (Bellis et al., 2014). Secondly, and most importantly for adult neurodevelopmental service design, higher ACE exposure was associated with diagnostic non-confirmation in the primary analysis: each additional reported ACE was associated with a 15% reduction in the adjusted odds of receiving a neurodevelopmental diagnosis, and the proportion diagnosed declined monotonically from 35.6% among those reporting no adversity to 6.2% among those reporting six or more ACEs. Thirdly, ACE scores were associated with greater depressive symptoms, anxiety symptoms and drug-related problems, but not with alcohol use, and were higher among participants screening positive for possible personality disorder, possible head injury and, in raw-score comparisons, possible mood disorder. Fourthly, when depressive symptom scores and personality disorder screening status were added to the adjusted model, the ACE estimate attenuated and was no longer statistically significant, indicating that the association between adversity and diagnostic outcome overlaps substantially with concurrent psychiatric symptom burden.

These findings should be interpreted as associations rather than evidence of causation. The study measured retrospective exposure to childhood adversity, not current trauma-related psychopathology, and it does not demonstrate that adversity caused the presenting symptoms, nor that trauma explained the referral in any individual case. The pattern is nonetheless clinically important because it shows that childhood adversity is not merely background information in this referral population. It is associated with the psychiatric symptom profile and the diagnostic outcome pattern observed after specialist assessment. The findings therefore support the argument that adversity history should be incorporated into adult ADHD and autism assessment as a routine component of formulation, differential diagnosis and post-assessment care planning.

### 4.2 ACE exposure in adult neurodevelopmental referral populations

The distribution of ACE scores in this cohort indicates that adults referred for ADHD or autism assessment frequently present with substantial histories of childhood adversity. The proportion reporting four or more ACEs (39.7%) was nearly five times the corresponding English national household survey estimate of 8.3% (Bellis et al., 2014), and the proportion reporting no adversity (19.4%) was well below the national estimate of 53.6%. This is consistent with the wider ACE literature, which has repeatedly demonstrated graded associations between childhood adversity and adult depression, anxiety, substance misuse, functional impairment and health service use (Felitti et al., 1998; Chapman et al., 2004; Dube et al., 2003; Gondek et al., 2021; Tan and Mao, 2023). The present study extends that literature into a clinically specific population that has received relatively little empirical attention: adults entering neurodevelopmental diagnostic pathways.

This is important because adult neurodevelopmental services do not assess a diagnostically homogeneous group. Referrals are generated by symptom descriptions, functional concerns, self-recognition, family reports, primary care suspicion and sometimes service availability, rather than by confirmed diagnostic status. The referred population therefore includes people with probable ADHD or autism, people with mixed neurodevelopmental and psychiatric presentations, people with adversity-related attentional or social difficulties, and people whose difficulties arise mainly from other mental health conditions. The high ACE exposure observed here suggests that trauma-informed assessment is not an optional adjunct in this setting, but part of the minimum clinical context needed to interpret symptoms accurately.

### 4.3 Demographic influences on ACE reporting

Female participants reported higher ACE scores than male participants, and ACE scores increased modestly with age. These effects were statistically significant but small, and the regression model explained less than five percent of the variance in ACE scores. The findings therefore should not be overinterpreted as indicating strong demographic determinism. Rather, they suggest that age and gender may influence either exposure to adversity, willingness to disclose adversity, referral pathways into assessment, or some combination of these mechanisms.

The higher ACE scores among female participants are compatible with wider evidence that some forms of childhood adversity, particularly sexual abuse and exposure to domestic or relational harm, may be differentially distributed by gender. However, disclosure effects are also plausible. Female patients may be more likely to report adverse experiences in clinical settings, whereas male patients may underreport abuse, neglect or household dysfunction because of stigma, minimization or gendered expectations concerning emotional disclosure. The age association is also open to several interpretations. Older participants may have had greater developmental distance from childhood events, allowing more coherent recall and disclosure, or may represent a subgroup whose difficulties remained unformulated until later adulthood. These interpretations remain speculative and should be tested in larger prospective datasets. The independent association between female gender and lower odds of diagnosis in the adjusted model is consistent with wider evidence concerning gender differences in adult neurodevelopmental diagnosis and warrants investigation in its own right.

### 4.4 Adversity and psychiatric symptom burden

The associations between ACE scores and depressive symptoms, anxiety symptoms and drug use were clinically coherent and consistent with previous ACE research. The strongest continuous association was with PHQ-9 depression scores, followed by GAD-7 anxiety scores and DAST-10 drug-use scores; given the strong intercorrelation of the PHQ-9 and GAD-7 (*r* = 0.78), the depression and anxiety associations are best understood as reflecting a shared internalizing symptom dimension rather than two independent effects. These findings support the interpretation of ACE exposure as a transdiagnostic marker of psychiatric complexity in adults referred for neurodevelopmental assessment. In practical terms, a high ACE score in this population should prompt careful formulation of mood symptoms, anxiety symptoms, substance use, relational instability, self-concept, emotion regulation and risk, rather than being treated as an incidental historical variable.

The association between ACE exposure and IPDS positivity is particularly relevant. The IPDS is a screening instrument rather than a diagnostic assessment for personality disorder, and its result should not be treated as a categorical diagnosis. Nevertheless, the pattern suggests that higher childhood adversity is associated with enduring interpersonal, affective and behavioral difficulties that may complicate neurodevelopmental assessment, a pattern consistent with the recognized overlap between ADHD and borderline personality features in adults (Weiner et al., 2019). In such cases, apparent social communication difficulty, emotional reactivity, executive dysfunction or occupational instability may reflect several interacting mechanisms. These may include ADHD, autism, adversity-related adaptation, personality-related vulnerability, mood or anxiety symptoms, substance use, head injury, or combinations of these. The high overall rate of IPDS positivity (68.7%) underlines the clinical heterogeneity of this referral population and may itself be relevant to diagnostic non-confirmation, since enduring interpersonal and affective difficulties of this kind can generate neurodevelopmental-like referral presentations; this possibility is consistent with the attenuation of the ACE estimate when IPDS status was added to the exploratory model.

The association between ACE exposure and HELPS positivity could suggest that possible head injury may be a consequence of childhood physical abuse, interpersonal violence, risk-taking behavior, accidents, or later-life adversity. It may also independently contribute to attentional difficulties, executive dysfunction, irritability and emotional dysregulation. This creates an additional differential diagnostic challenge, because acquired neurocognitive impairment can resemble or amplify ADHD-like symptoms. Notably, no participant reporting zero ACEs screened positive on the HELPS. Future studies should distinguish self-reported head injury screens from clinically confirmed traumatic brain injury and should examine whether head injury mediates any relationship between ACE exposure and neurodevelopmental-like symptom presentation.

The absence of a significant association between ACE scores and AUDIT alcohol-use scores differs from much of the broader ACE literature. Several explanations are possible. Alcohol consumption may have been underreported in the context of assessment for ADHD, particularly where patients were aware that substance use can affect prescribing decisions. Alternatively, the AUDIT may not capture the most clinically relevant alcohol-related patterns in this specific population, or drug use may be a more sensitive marker of adversity-linked coping behavior in this cohort. The null finding should therefore be reported as an empirical result, not as evidence that ACEs are unrelated to alcohol misuse in adult neurodevelopmental referral populations generally.

### 4.5 Diagnostic outcome and childhood adversity: candidate explanations

The most clinically important finding is the association between higher ACE exposure and diagnostic non-confirmation. This may appear counterintuitive because neurodevelopmental conditions can increase vulnerability to adversity, bullying, exploitation, social exclusion and repeated failure experiences. Under a double jeopardy model (Gajwani and Minnis, 2023), one might expect diagnosed participants to show higher ACE exposure. The observed pattern was the opposite: very high ACE exposure was more common in those who did not receive ADHD or autism diagnoses. Because the design is cross-sectional and the study did not measure trauma-related psychopathology, this association admits several candidate explanations, which cannot be adjudicated with the present data and are therefore set out explicitly.

First, adversity-related psychopathology may produce presentations that resemble ADHD or autism at the level of observable symptoms but arise from different developmental mechanisms, a possibility we term the trauma-related phenocopy hypothesis and advance strictly as a hypothesis for future research rather than as an explanation of the present findings. On this hypothesis, childhood adversity could produce attentional difficulties through hypervigilance, intrusive memories, dissociation, sleep disturbance, anxiety and impaired concentration; ADHD-like emotional dysregulation through altered threat processing, low frustration tolerance and reactive impulsivity; and autism-like social withdrawal through attachment disruption, shame, avoidance, mistrust, reduced social learning opportunities and defensive interpersonal strategies. Comparable overlap between post-adversity phenotypes and ADHD has been described in children (Lazzaro et al., 2025), and case-level accounts illustrate the difficulty of distinguishing autism from the consequences of early traumatisation during diagnostic assessment (Kildahl et al., 2024). The present study did not measure PTSD, complex PTSD, dissociation or attachment, and a high ACE score cannot be equated with trauma-related symptomatology; direct measurement of these constructs is required before the hypothesis can be tested.

Secondly, the association may be carried by concurrent psychiatric symptom burden rather than by adversity as such. The exploratory model is consistent with this: when depressive symptoms and personality disorder screening status were added, the ACE estimate attenuated toward the null. Cross-sectional data cannot distinguish whether psychiatric symptoms mediate an effect of adversity, confound it, or share common causes with it. Thirdly, reverse and bidirectional pathways must be taken seriously: neurodevelopmental difficulties themselves increase exposure to bullying, family conflict, social exclusion, exploitation and educational adversity, and shared familial and socioeconomic factors may underlie both adversity and referral. Fourthly, reporting and ascertainment mechanisms may contribute, including differential disclosure of adversity, and the fact that clinicians had access to screening results, including the ACE-Q, during assessment, which creates the possibility of incorporation bias whereby knowledge of adversity influences diagnostic formulation. The present data cannot distinguish among these explanations, and the causal language throughout this paper is correspondingly conservative.

The pathway-specific analyses do not alter this picture. When ACE score was analyzed continuously, the association with diagnostic outcome was similar in direction and magnitude in both pathways; the categorical analysis reached significance only in the autism pathway, but subgroup sizes were small, expected cell counts were low and the confidence intervals for the effect sizes overlapped substantially. These results provide no reliable evidence that the association is specific to either pathway, and no such claim is made.

### 4.6 Implications for adult ADHD and autism assessment

The findings support routine enquiry about childhood adversity within adult ADHD and autism assessment, but they do not support using ACE scores as an exclusionary gatekeeping tool. This distinction is essential. A high ACE score should not be interpreted as evidence against ADHD or autism, because childhood adversity and neurodevelopmental conditions can and do co-exist; the double jeopardy literature shows that neurodevelopmental vulnerability may increase exposure to adversity. Co-occurrence is common and clinically important, and adversity and neurodevelopmental diagnosis should not be framed as competing explanatory models. Conversely, a low ACE score does not confirm a neurodevelopmental condition. ACE screening should therefore inform clinical formulation, not replace diagnostic assessment.

In practice, ACE screening may improve assessment quality in several ways. It can identify adversity-related mechanisms that require direct exploration during clinical interview. It can guide differential diagnosis by encouraging clinicians to examine onset, developmental persistence, cross-situational consistency, adversity chronology, episodic vs. lifelong patterning, and the presence or absence of early childhood neurodevelopmental markers. It can also support safer clinical management by identifying patients who may need trauma-informed communication, pacing of assessment, safeguarding consideration, risk assessment or onward referral for trauma-focused intervention, consistent with the wider literature on trauma-informed practice in neurodevelopmental assessment (Peterson et al., 2019; Langenfeld et al., 2021; Michna et al., 2023).

The findings also have implications for the interpretation of self-report screening questionnaires. Adult neurodevelopmental services often receive referrals supported by high scores on ADHD or autism screening instruments. However, screening tools are vulnerable to non-specific symptom inflation in populations with depression, anxiety, adversity histories, substance use, personality-related difficulties and acquired cognitive problems. A trauma-informed pathway should therefore avoid treating questionnaire elevation as equivalent to diagnostic probability. Screening results should be interpreted alongside developmental history, informant evidence where available, functional impairment, symptom chronology, differential diagnoses and clinical observation.

### 4.7 Service and commissioning implications

The results have service-level relevance because adult neurodevelopmental pathways are under sustained demand pressure. If a substantial subgroup of referred adults have clinically significant adversity-related or psychiatric difficulties but do not meet ADHD or autism criteria, services need pathways that can respond constructively after diagnostic non-confirmation. Without such pathways, non-diagnosis may be experienced by patients as dismissal, even when the assessment is clinically sound. This creates risk for dissatisfaction, repeated referrals, complaint escalation and continued unmet need.

A trauma-informed service model would not simply add an ACE questionnaire to existing processes. It would require a pathway architecture in which adversity history is considered at referral review, triage, assessment, formulation, feedback and signposting stages. Patients whose symptoms are not best explained by ADHD or autism should receive a clear explanation of the differential diagnostic reasoning and, where appropriate, signposting to trauma-focused psychological therapy, community mental health services, substance-use services, brain injury services, primary care mental health support or social prescribing. This is not an argument for diverting patients away from neurodevelopmental assessment solely because they report adversity. It is an argument for ensuring that adult ADHD and autism services are not left as the default destination for all unresolved attentional, emotional and social difficulties.

The findings also suggest that commissioning models should recognize the clinical heterogeneity of neurodevelopmental referral populations. A binary model in which patients either receive a neurodevelopmental diagnosis or are discharged without meaningful formulation is unlikely to meet the needs of this group. Commissioners should consider integrated models that include diagnostic assessment, differential formulation, trauma-informed triage, psychiatric screening and post-assessment pathways for non-diagnosed patients whose difficulties remain clinically significant.

### 4.8 Strengths and limitations

The study has several strengths. It used data from a real-world NHS adult ADHD and autism service, making the findings directly relevant to routine clinical practice. The sample included both ADHD and autism pathways, allowing examination of whole-cohort and pathway-specific patterns. Diagnostic outcomes were based on full specialist multidisciplinary assessment rather than screening scores alone. The analysis incorporated multiple clinical screening instruments, permitting examination of ACE exposure in relation to depressive, anxiety and substance use symptoms and to mood disorder, head injury and personality disorder screening status. The availability of near-complete screening data for cases awaiting an outcome permitted a direct assessment of selection into the analytic sample.

The limitations are also substantial. The retrospective cross-sectional design prevents causal inference and does not establish temporal mechanisms beyond the historical nature of ACE reporting. The analytic sample comprised 60.1% of eligible cases, because 38.4% of the source cohort had not yet received a diagnostic outcome at data extraction. Although included cases did not differ from cases awaiting an outcome on any measured characteristic (Table 1), selection on unmeasured characteristics cannot be excluded, and the findings describe adults who completed assessment during the study window rather than all adults referred to the service. The study was conducted in a single regional NHS service, which may limit generalisability to other services, private-sector providers, different geographical areas or healthcare systems with different referral thresholds.

ACEs were assessed by self-report and may be affected by recall bias, current mood state, stigma, dissociation, minimization or differences in willingness to disclose adversity. The ACE-Q captures exposure categories rather than severity, chronicity, timing, perpetrator relationship, subjective impact or protective factors, and two individuals with identical scores may have experienced markedly different adversity; for this reason the manuscript refers throughout to ACE exposure rather than trauma burden. The study did not include structured measures of PTSD or complex PTSD, which limits the ability to distinguish ACE exposure from current trauma-related disorder and precludes any direct test of trauma-related mechanisms.

The comorbidity-related instruments used in this study are screening tools and are not diagnostic assessments; positive screens on the MDQ, HELPS or IPDS indicate possible clinical concerns requiring further assessment, and the manuscript accordingly describes psychiatric symptom burden and positive screening status rather than confirmed psychiatric comorbidity. Because the screening instruments were administered as part of the diagnostic pathway and their results, including the ACE-Q, were available to assessing clinicians, two related biases must be acknowledged: incorporation bias, whereby knowledge of adversity or psychiatric symptoms may have influenced diagnostic formulation, and partial circularity between the exposure-related analyses and the diagnostic decision process. These do not invalidate the observed associations, which concern routinely collected information as actually used in the service, but they qualify any mechanistic interpretation. Relatedly, the study cannot establish diagnostic accuracy in any formal sense, because no external gold standard exists against which the specialist diagnostic decisions could be validated; diagnostic outcome here means the outcome of specialist assessment, not an independently verified diagnostic truth.

The diagnosed group was relatively small (47 cases), which constrained the number of covariates in the logistic regression and reduced the precision of pathway-specific estimates. The exploratory six-predictor model falls below conventional events-per-predictor guidance and is interpreted accordingly. Multiple statistical tests were performed across the secondary analyses, which increases the risk of type I error; the diagnostic outcome analysis was designated primary, all other analyses are labeled exploratory, and no formal multiplicity correction was applied to them. Although the most coherent findings were consistent across related clinical domains and are supported by confidence intervals rather than isolated *p*-values, replication is required, and several statistically significant effects were small in absolute terms; statistical significance should not be read as clinical significance.

Another limitation concerns diagnostic outcome classification. The analysis compared diagnosed and non-diagnosed participants, but did not examine subgroups within the non-diagnosed category. Some non-diagnosed patients may have had adversity-related presentations, while others may have had anxiety disorder, depressive disorder, personality-related difficulties, substance use, acquired cognitive impairment, subthreshold neurodevelopmental traits, insufficient developmental evidence, or mixed formulations. Future work should examine clinician-rated final formulations rather than diagnostic outcome alone.

### 4.9 Future research

Prospective multi-site research is needed to test whether these findings replicate across adult ADHD and autism services with different referral thresholds, assessment models and population characteristics. Future studies should include structured measures of PTSD and complex PTSD, clinician-rated trauma formulation, informant developmental history, functional impairment measures and longitudinal follow-up. This would permit a direct test of the trauma-related phenocopy hypothesis and examination of whether high ACE exposure predicts diagnostic non-confirmation independently of depression, anxiety, substance use, head injury and personality-related difficulty, which the present exploratory analysis could not establish.

Future work should also examine whether trauma-informed triage improves the quality of diagnostic formulation, patient experience, waiting-list management and post-assessment outcomes. A particularly important question is whether patients with high ACE exposure and non-confirmed neurodevelopmental diagnosis benefit from targeted trauma-informed interventions after assessment. Longitudinal studies could determine whether attentional, emotional and social difficulties improve following trauma-focused treatment, remain stable as enduring neurodevelopmental traits, or require integrated intervention addressing both adversity-related and neurodevelopmental needs.

## 5 Conclusion

Adults completing ADHD and autism assessment in this regional NHS service reported substantially higher ACE exposure than English population estimates. Higher ACE exposure was associated with diagnostic non-confirmation, before and after adjustment for age, gender and pathway, and with greater psychiatric symptom burden across depressive, anxiety, drug use and screening measures. These findings support consideration of childhood adversity as part of trauma-informed formulation during adult neurodevelopmental assessment. They do not demonstrate that trauma-related mechanisms caused the presentations observed, and the association with diagnostic outcome was not independent of concurrent psychiatric symptom burden in exploratory analysis. Prospective studies incorporating direct measures of trauma-related psychopathology are needed to determine whether trauma-related mechanisms contribute to neurodevelopmental-like presentations. The findings are associative and largely exploratory, and replication is required before pathway-level conclusions can be drawn.

## Data Availability

The data analysed in this study were derived from routinely collected clinical records. Because of the clinical nature of the dataset and the risk of re-identification, individual-level data are not publicly available. Aggregated data may be available from the corresponding author subject to appropriate governance approvals. Requests to access these datasets should be directed to sarah.jones1@swyt.nhs.uk.
