# Supplementary material for: Adverse childhood experiences in adults completing ADHD and autism diagnostic assessment: associations with diagnostic outcome and psychiatric symptom burden
Source: Front Psychol. 2026 Sep 17;17:1898116. doi: 10.3389/fpsyg.2026.1898116 (PMC13626892; doi:10.3389/fpsyg.2026.1898116)
Supplement: Supplementary file 1 [file Supplementary_file_1.docx]

**Supplementary material: STROBE checklist for cross-sectional studies**

Manuscript ID 1898116. Item locations refer to sections of the revised manuscript.

| **Item** | **Recommendation** | **Location in revised manuscript** |
| --- | --- | --- |
| 1a | Title and abstract: indicate the study design | Abstract (retrospective cross-sectional cohort study); title identifies population and variables |
| 1b | Provide an informative and balanced summary | Abstract |
| 2 | Background/rationale | Introduction, paragraphs 1-8 |
| 3 | State specific objectives, including any prespecified hypotheses | Introduction, final paragraph |
| 4 | Present key elements of study design early | Section 2.1 |
| 5 | Describe the setting, locations and relevant dates | Section 2.1 |
| 6a | Give the eligibility criteria and the sources and methods of selection | Sections 2.1 and 2.2; Figure 1 |
| 7 | Clearly define all outcomes, exposures, predictors, potential confounders and effect modifiers | Sections 2.2, 2.3 and 2.5 |
| 8 | Give sources of data and details of methods of assessment for each variable | Section 2.3 (2.3.1-2.3.8) |
| 9 | Describe any efforts to address potential sources of bias | Sections 2.2 (included versus awaiting comparison), 2.5, 4.5 and 4.8 (selection, incorporation and circularity biases) |
| 10 | Explain how the study size was arrived at | Section 2.2; Figure 1 (all eligible cases in the study period) |
| 11 | Explain how quantitative variables were handled, and why | Sections 2.3.8 and 2.5 (continuous primary analysis; descriptive categories justified) |
| 12a | Describe all statistical methods, including those used to control for confounding | Section 2.5 |
| 12b | Describe any methods used to examine subgroups and interactions | Sections 2.5 and 3.3 (pathway-specific analyses) |
| 12c | Explain how missing data were addressed | Sections 2.5 and 3.1 (available-case analysis with full reporting) |
| 12d | If applicable, describe analytical methods taking account of sampling strategy | Not applicable (whole-service consecutive sample) |
| 12e | Describe any sensitivity analyses | Section 2.5 and 3.3 (inconclusive cases recoded; exploratory six-predictor model) |
| 13a | Report numbers of individuals at each stage of the study | Section 3.1; Figure 1 |
| 13b | Give reasons for non-participation at each stage | Section 2.2; Figure 1 |
| 13c | Consider use of a flow diagram | Figure 1 |
| 14a | Give characteristics of study participants and information on exposures and potential confounders | Table 1 |
| 14b | Indicate number of participants with missing data for each variable | Table 1 (n column); Section 3.1; Figure 1 |
| 15 | Report numbers of outcome events or summary measures | Sections 3.1 and 3.3; Tables 1 and 4; Figures 2 and 3 |
| 16a | Give unadjusted estimates and, if applicable, confounder-adjusted estimates and their precision; make clear which confounders were adjusted for and why | Section 3.3; Table 4 |
| 16b | Report category boundaries when continuous variables were categorised | Section 2.3.8 |
| 16c | If relevant, consider translating estimates of relative risk into absolute risk | Section 3.3 and Figure 3 (absolute proportions diagnosed by ACE category) |
| 17 | Report other analyses done, e.g. subgroup and sensitivity analyses | Sections 3.2, 3.3 and 3.4 |
| 18 | Summarise key results with reference to study objectives | Section 4.1 |
| 19 | Discuss limitations, taking into account sources of potential bias or imprecision | Section 4.8 |
| 20 | Give a cautious overall interpretation of results | Sections 4.5 and 5 |
| 21 | Discuss the generalisability of the study results | Section 4.8 |
| 22 | Give the source of funding and the role of the funders | Funding statement (no funding received) |
